# Graphitic Carbon Nitride Nanomaterials-Based Electrochemical Sensing Interfaces for Monitoring Heavy Metal Ions in Aqueous Environments

**DOI:** 10.3390/nano15070564

**Published:** 2025-04-07

**Authors:** Cheng Yin, Yao Liu, Tingting Hu, Xing Chen

**Affiliations:** 1 School of Resources and Environmental Engineering, Anhui Water Conservancy Technical College, Hefei 231603, China; yc@ahsdxy.edu.cn; 2 School of Resources and Environmental Engineering, Hefei University of Technology, Hefei 230009, China; htting0212@163.com; 3 Key Lab of Aerospace Structural Parts Forming Technology and Equipment of Anhui Province, Institute of Industry and Equipment Technology, Hefei University of Technology, Hefei 230009, China

**Keywords:** g-C_3_N_4_, g-C_3_N_4_-based nanomaterials, heavy metal ions, electrochemical detection, environmental monitoring

## Abstract

The persistent threat of heavy metal ions (e.g., Pb^2+^, Hg^2+^, Cd^2+^) in aqueous environments to human health underscores an urgent need for advanced sensing platforms capable of rapid and precise pollutant monitoring. Graphitic carbon nitride (g-C_3_N_4_), a metal-free polymeric semiconductor, has emerged as a revolutionary material for constructing next-generation environmental sensors due to its exceptional physicochemical properties, including tunable electronic structure, high chemical/thermal stability, large surface area, and unique optical characteristics. This review systematically explores the integration of g-C_3_N_4_ with functional nanomaterials (e.g., metal nanoparticles, metal oxide nanomaterials, carbonaceous materials, and conduction polymer) to engineer high-performance sensing interfaces for heavy metal detection. The structure-property relationship is critically analyzed, emphasizing how morphology engineering (nanofibers, nanosheets, and mesoporous) and surface functionalization strategies enhance sensitivity and selectivity. Advanced detection mechanisms are elucidated, including electrochemical signal amplification, and photoinduced electron transfer processes enabled by g-C_3_N_4_’s tailored bandgap and surface active sites. Furthermore, this review addresses challenges in real-world deployment, such as scalable nanomaterial synthesis, matrix interference mitigation, and long-term reliable detection. This work provides valuable insights for advancing g-C_3_N_4_-based electrochemical sensing technologies toward sustainable environmental monitoring and intelligent pollution control systems.

## 1. Introduction

The contamination of water pollution has become a matter of public concern in environmental issues, especially heavy metal ions (HMIs), which can result in serious injury to public health even at very low levels of exposure [1,2]. Simultaneously, the enrichment and transmission of toxic metals in the food and food chain is one of the main problems in ecology [3,4]. Sources of heavy metal ions include industrial and municipal effluent discharges, as well as mining and smelting. Trace heavy metals such as zinc, copper, and cobalt are essential trace elements for the human body; while others, such as cadmium, mercury, and lead, are highly toxic and cause irreversible damage to the human body [5,6]. In addition, the toxicity of heavy metal pollutants also depends on their main form. For example, Cr(III) is essential for most biological systems, and Cr(VI) is a highly toxic substance that can cause kidney failure, nervous system damage, and even death [7]. The permitted threshold concentrations for common heavy metal ions in drinking water, as established by major regulatory bodies such as the U.S. Environmental Protection Agency (EPA) and the World Health Organization (WHO), are as follows: lead (Pb) < 5 μg/L; mercury (Hg) < 2 μg/L; cadmium (Cd) < 5 μg/L; arsenic (As) < 10 μg/L; chromium (Cr) < 50 μg/L [8]. Thus, establishing a sensitive, specific, and fast detection method for heavy metal ions is essential to prevent their ecotoxicological hazards.

At present, various methods, such as atomic absorption spectroscopy (AAS) [9], ultraviolet-visible spectrophotometry (UV) [10], X-ray spectroscopy (XRF) [11,12], and inductively coupled plasma mass spectrometry (ICP-MS) [13], have been used for the determination of heavy metal ions. Among these methods, the electrochemical method is favored by researchers for its low cost, easy operation, high stability and sensitivity, and great application prospects [14]. These costly methods demand skilled operators, complex equipment, and multi-step preparation. Limited to quantification, they require chromatography integration for speciation analysis, risking sample alteration during handling/storage [15]. Therefore, developing rapid, low-cost, simple, and reliable technologies for in situ and real-time measurement of heavy metal ions remains an ongoing research focus.

Electrochemical detection methods, with their high sensitivity, simplicity, cost-effectiveness, and ease of integration, have attracted significant attention for enabling rapid on-site analysis of heavy metals in environments [16]. Common techniques include linear sweep voltammetry, cyclic voltammetry, stripping voltammetry, and chronoamperometry. The presence of modified materials for the electrode plays an essential role in determining the detection sensitivity of the electrochemical sensors. In previous research, various available materials such as precious metals (Au, Ag, etc.) [17,18], transition metal oxides (TiO_2_, Fe_3_O_4_, Cu(OH)_2_, etc.) [19,20,21], g-C_3_N_4_ [22,23,24], graphene [25,26], and their nanostructured assemblies [27,28] have been extensively used as electrocatalysts to enhance redox reactions of reactive pollutants through adsorption and catalysis. Among them, graphitic carbon nitride (g-C_3_N_4_) is a metal-free polymer with good resistance to acid, alkalis, and high temperatures because of the strong covalent bonds between carbon and nitrogen atoms [29]. Compared with other materials, pure g-C_3_N_4_ has defects of a small specific surface area, low charge mobility, and low utilization of visible light. It is usually modified by doping and changing its morphology to improve the specific surface area, enhance the charge migration, and improve the utilization of visible light [30,31]. In the field of environment and energy, g-C_3_N_4_ can be used for the photocatalytic degradation of pollutants [32,33], photocatalytic hydrogen production [34,35], carbon dioxide reduction [36,37], supercapacitors [38,39], and the adsorption of heavy metal ions [40]. In recent years, g-C_3_N_4_ and the nanocomposites based on g-C_3_N_4_ have been favored by a large number of researchers and also have been applied in electrochemical sensors for detecting heavy metal ions.

Although some review papers have introduced g-C_3_N_4_-based electrochemical sensors for environmental pollutant sensing [31,41], it is crucial to summarize the characteristics of g-C_3_N_4_-based electrochemical sensors in detecting heavy metal contaminants by analyzing the sensing behavior at sensitive interfaces and the morphological modulation of g-C_3_N_4_. Consequently, this review summarizes the detection mechanism of g-C_3_N_4_ and the properties of g-C_3_N_4_; the potential application of g-C_3_N_4_ in heavy metal detection was discussed and prospected. It is hoped that this article can provide some help to the majority of researchers, to make greater breakthroughs and innovations in future research.

## 2. The Performance Characteristics of C_3_N_4_

### 2.1. The Crystal Structure of C_3_N_4_

**Figure 1:**
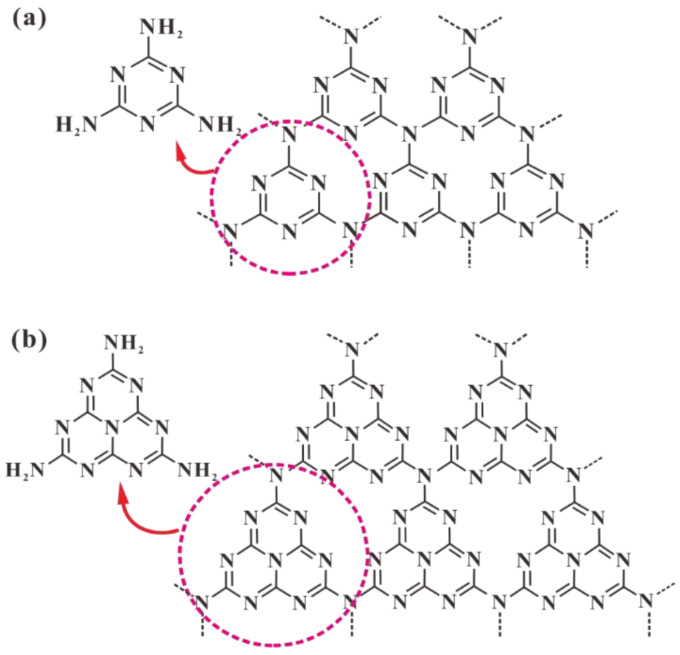
s-Triazine- (**a**) and tri-s-triazine-based (**b**) structures of g-C_3_N_4_ [48].

The crystal structure model of C_3_N_4_ was established based on first principles, including the α phase (α-C_3_N_4_), β phase (β-C_3_N_4_), cubic phase (c-C_3_N_4_), quasicubic phase (q-C_3_N_4_) and graphite-like phase (g-C_3_N_4_) [42,43,44]. As shown in Figure 1 below, the structure of the graphite-like phase (g-C_3_N_4_) is the most stable allotrope of carbon nitride and has attracted much attention in recent years [45]. It has a layered structure similar to graphite and contains two allotropes, namely tri-s-triazine (C_6_N_7_) and s-triazine (C_3_N_3_) rings. The s-triazine ring is aromatic, so the conjugated two-dimensional polymer of s-triazine will tend to form a p-conjugated plane layer, similar to graphite. Among them, tri-s-triazine is more stable in thermodynamics. Research has revealed that different crystal structures of C_3_N_4_ may significantly influence its electrochemical sensing performance. For instance, the α-phase demonstrates higher conductivity compared to the β-phase, which can be attributed to the more delocalized π-electrons in the α-phase that facilitate faster electron transfer during redox reactions [46]. The two-dimensional layered structure of graphitic-phase C_3_N_4_ (g-C_3_N_4_) promotes ion diffusion and migration, thereby exhibiting superior electrochemical performance in heavy metal detection [47]. These structure-property relationships may provide valuable guidance for material selection in specific sensing applications.

### 2.2. The Electronic Properties of C_3_N_4_

**Figure 2:**
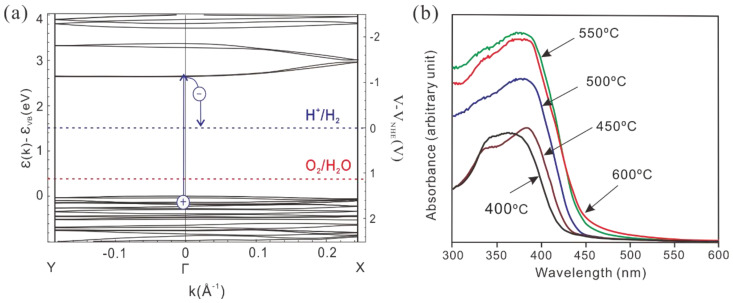
(**a**) Density-functional−theory band structure for polymeric melon calculated along the chain (Γ–X direction) and perpendicular to the chain (Y–Γ direction). (**b**) Ultraviolet−visible diffuse reflectance spectrum of carbon nitrides prepared at different temperatures [52].

By contrast with traditional semiconductors (e.g., TiO_2_ and ZnO), g-C_3_N_4_ owns a two-dimensional (2D) lamellar structure with a π conjugated system and a moderate energy gap (∼2.7 eV) [14], which lead to unique optoelectronic properties, high chemical stability, and good visible-light absorption (approximately at 450∼460 nm). The plane of g-C_3_N_4_ consists of sp^2^ hybridized CN aromatic heterocycles. Among them, atoms in the layer are arranged in a honeycomb structure with strong covalent bonds and van der Waals forces in between layers. The valence band edge of g-C_3_N_4_ is mainly composed of N 2p; thus, the photogenic holes are generated at the N position. The conduction band edge is composed of N 2p and C 2p state hybridization, so the photogenerated electron bears high binding energy in the electron-hole pair, which leads to the high photogenerated charge carrier recombination rate of g-C_3_N_4_ [49,50,51]. Studies have shown that the band structure of the polymer melon structure is non-isotropic, in which there is a direct band gap at Γ and only dispersion in the Γ–X direction (Figure 2a). Furthermore, different thermal condensation temperatures will affect the electronic and optical properties of the synthesized carbon nitride [52]. The absorption edge shifts to longer wavelengths, indicating that the band gap decreases as the condensation temperature increases (Figure 2b).

### 2.3. Surface Physicochemical Properties of C_3_N_4_

**Figure 3:**
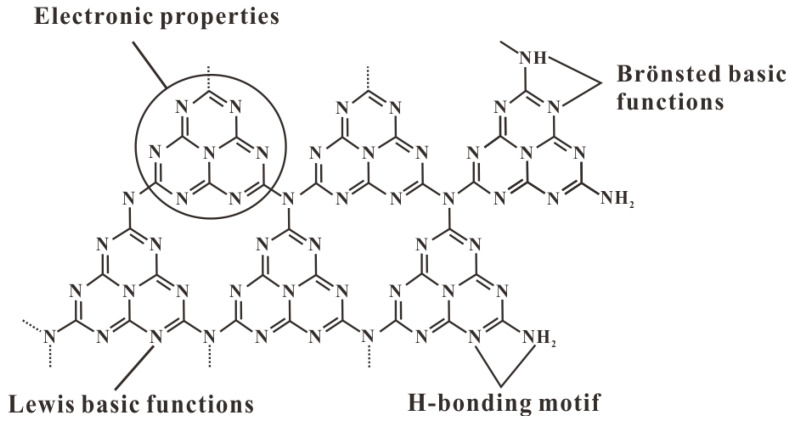
Multiple functionalities of C_3_N_4_ as a catalyst [61].

Compared with most carbon materials, g has electron-rich properties, basic surface functional groups, and H-bonding motifs due to the presence of N and H atoms. Therefore, it is considered a potential candidate to supplement carbon in material applications [53,54,55]. Due to the influence of incomplete polycondensation, there are some defects on the surface of g-C_3_N_4_, namely the basic primary and/or secondary amine groups at the end of g-C_3_N_4_ (Figure 3). Compared with the ideal defect-free g-C_3_N_4_, g-C_3_N_4_ with surface defects has excellent electron-rich properties, leading to the formation of a variety of functions. In addition, the abundant basic functional groups on the surface of g-C_3_N_4_ can absorb acidic and toxic molecules through electrostatic action [55]. However, the hydrophobicity of g-C_3_N_4_ leads to weak interfacial interactions, which further inhibit surface electron transfer and catalytic reactions [56]. To address this issue, materials such as nonmetallic elements, metal nanoparticles, carbon nanotubes, and graphene-based flakes can be doped between the layers of g-C_3_N_4_ to significantly enhance its hydrophilicity [57]. Zhu et al. [58]. in situ grew CeO_2_ nanocrystals on the surface of two-dimensional MoS_2_/g-C_3_N_4_ nanosheets. The ternary composite material significantly enhanced the hydrophilicity of the substrate. In addition, the hydrophilicity can be improved by introducing oxygen-containing functional groups on the g-C_3_N_4_ structure, which can enhance the interfacial coupling and photocatalytic activity [59]. Shen et al. [60] introduced π-electron-rich domains and hydrophilic hydroxyl groups into the g-C_3_N_4_ structure to form tartaric acid-modified g-C_3_N_4_ (TA-CN). In this system, the hydroxyl groups regulate reaction kinetics through their hydrophilicity, enhance the selective adsorption of reactants, and stabilize intermediates.

### 2.4. Photoelectrochemical Properties of C_3_N_4_

Graphitic carbon nitride (g-C_3_N_4_) is defined as an emerging semiconductor for applications in photocatalytic and photoelectrochemical analysis. As a metal-free photocatalyst, g-C_3_N_4_ has been shown to possess the band characteristics of metal oxide semiconductors with a band gap of about 2.7 eV and spectral response up to 460 nm, indicating visible light absorption [62]. Thus, g-C_3_N_4_ is regarded as a promising semiconductor for light-emitting devices, photocatalysis, photoelectrodes, energy conversion, photodegradation, and PEC sensors [63,64,65,66]. This work was proposed by Du et al. who synthesized 3D branched crystalline carbon nitride with 1D nanoneedles [67]. It has a high specific surface area, fast photo-generated charge transfer, and excellent light-gathering performance. More importantly, the electron-hole pair separation and crystallinity are improved. In addition, Bu et al. proved the photocurrent conversion performance and photocathodic protection performance of g-C_3_N_4_ in 304 stainless steel under the irradiation of visible light or white light [68]. Su et al. used mpg-C_3_N_4_ as a photocatalyst, activated O_2_ under visible light irradiation, and achieved high selective catalysis of benzaldehyde [69]. In addition, the photocatalytic performance of visible light-sensitive metal-free mesoporous graphite carbon nitride can be used to establish a system to degrade drugs for the treatment of cardiovascular diseases [70].

### 2.5. Electrochemical Properties of C_3_N_4_

As a multifunctional electrocatalyst, the g-C_3_N_4_ semiconductor is useful in electrochemical detection and biosensing due to its high activity, large surface area, and fast electron transfer rate. Among the multifunctional electrocatalysts, pyridine N atoms in g-C_3_N_4_ with strong electron acceptors are considered as the active sites for electrochemical reactions [71]. g-C_3_N_4_ has been used in electrochemical sensing applications in the analysis of water environment pollutants. For example, g-C_3_N_4_ ultrathin nanosheets displayed higher surface reactive sites and better electrical conductivity and exhibited good selectivity and repeatability in Cd^2+^ detection [24,72]. In addition, the electrochemical sensing device based on g-C_3_N_4_ can also achieve biomarker analysis. g-C_3_N_4_ was used in the development of a glucose sensor as well as for the detection of dopamine and H_2_O_2_. Furthermore, g-C_3_N_4_ has been utilized in DNA analysis through stacked g-C_3_N_4_ nanofibers modified sensor detection of free DNA bases, cell analysis in the area of recognition and quantification of specific cells, analysis of heavy metals ions, as well as the detection of high-energy explosives and nerve agents.

## 3. Electrochemical Detection of Heavy Metal Ions

### 3.1. Electrochemical Detection Methods

**Figure 4:**
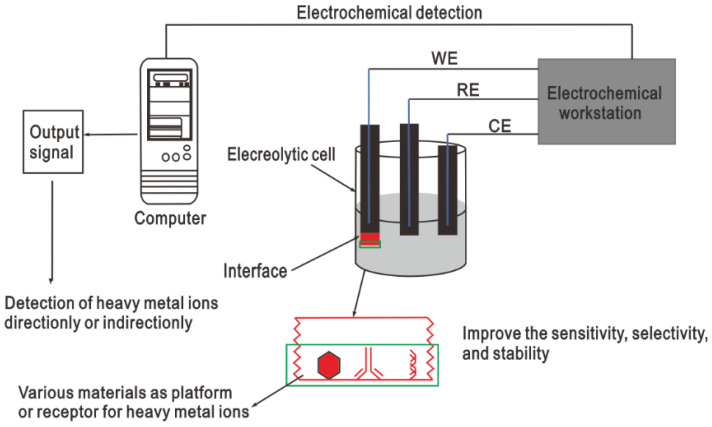
Schematic illustration of the general principle of electrochemical sensing of heavy metal ions [77].

The electrochemical detection method has a short analysis time, low power cost, and high sensitivity, and can be detected in situ [73]. Therefore, it has aroused great interest in the detection of heavy metal ions. Electrochemical sensing of HMI involves the use of biosensing electrodes that are employed to pass current to the aqueous solution and generate some useful and measurable electrical signal corresponding to the electrochemical reactions within the solution due to the presence of metal ions. The general experimental device for electrochemical detection of heavy metal ions usually consists of an electrolytic cell composed of an ion conductor (electrolyte) and an electronic conductor (electrode) [74]. In this case, the aqueous solution composed of HMI acts as an electrolyte. The battery potential is measured at the interface between the electrode and the electrolyte solution. Usually, electrochemical detection uses a three-electrode system, including a working electrode (WE), a reference electrode (RE), and a counter electrode (CE) (Figure 4). In the electrochemical detection process, different types of electrochemical techniques are classified according to the different types of detection signals generated by the presence of heavy metal ions in the solution matrix, such as current, potential, conductivity, electrochemical impedance, electrochemiluminescence, etc. [75,76]. Through customized functionalization and assembly, these materials can be easily assembled on the electrode surface for the manufacture of sensing electrodes and the sensitive and selective detection of heavy metal ions. The assembly of different materials such as metals, metal films, metal oxides, nanomaterials, carbon nanotubes, polymers, microspheres, and biological materials can further improve electrochemical performance. Based on these detection signals, electrochemical sensing can be divided into the amperometric method, potentiometric method, electrochemical impedance method, capacitance method, and electrochemiluminescence method. In most of these techniques, the changes in other parameters are measured by controlling any of the current or electric potentials.

### 3.2. Possible Mechanisms of Heavy Metal Ion Detection Based on g-C_3_N_4_ Nanomaterials

**Figure 5:**
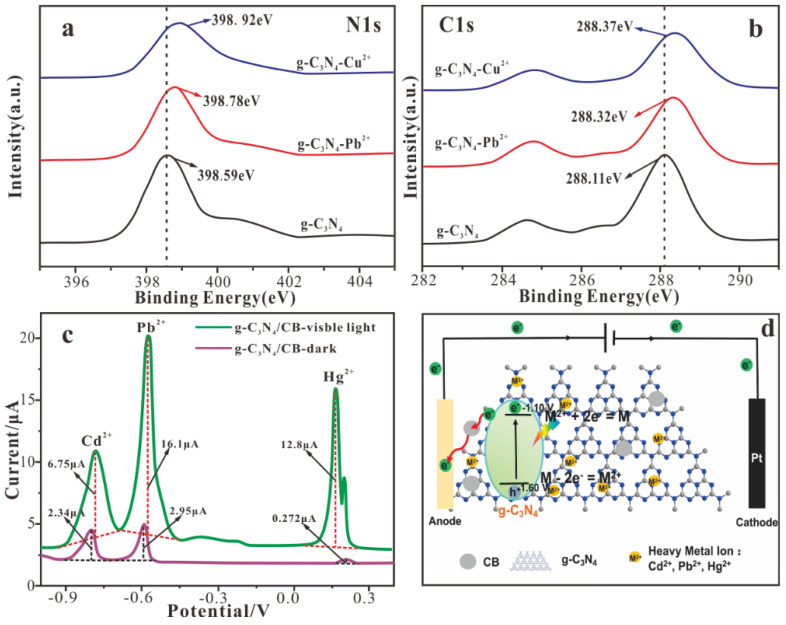
XPS high-resolution spectra of N 1s (**a**) and C 1s (**b**) before and after the adsorption of heavy metal ions (Pb(II) and Cu(II)). (**c**) DPASV curves of Cd^2+^, Pb^2+,^ and Hg^2+^ at g-C_3_N_4_/CB electrode under dark and visible light irradiation. (**d**) Scheme of the photo−assisted electrochemical detection of heavy metal ions of g-C_3_N_4_/CB electrode [81].

Stable and reliable heavy metal ion response signals are an important guarantee for electrochemical analysis methods to meet actual application requirements. The focus is on exploring the interaction between heavy metal ion forms and electrode modification materials, as well as their diffusion and transformation processes on sensitive interfaces. In the detection process of heavy metal ions, good adsorption performance can significantly improve the electrochemical detection performance. Based on this, nanomaterials with high specific surface area, excellent catalytic activity, electrical conductivity, and surface modifiability show significant advantages in electrochemical detection [78]. While a large number of binding sites and functional groups dominate the high sorption capacity of g-C_3_N_4_ and g-C_3_N_4_-based materials through the formation of strong surface complexes [79]. The response mechanism of heavy metal ions was studied in detail by X-ray photoelectron spectroscopy. Figure 5 shows that C≡N and N–H functional groups may interact with Pb (II) and Cu (II) to form complexes. The delocalized π-electron system of the triazine ring (C_3_N_3_) and heptazine ring (C_6_N_7_) can act as Lewis bases, and metal ions act as Lewis acids. Therefore, through the Lewis acid−base interaction, the adsorption of heavy metal ions on the g-C_3_N_4_ is also ascribed to the strong surface complexation between the g-C_3_N_4_ and metal ions [80]. Figure 5a,b also shows that Cu(II) can form a complex with the g-C_3_N_4_ more powerfully than Pb(II).

The essence of electrochemical detection is the electrocatalytic reaction process at the sensing interface, which will affect the enrichment/reduction of heavy metals and the corresponding dissolution/oxidation signal. Therefore, elucidating the electrocatalytic mechanism between the sensing interface and the target pollutant is essential for the development of an effective functional sensing interface to achieve high sensitivity and selective detection of heavy metals. Hu et al. [81] prepared a metal-free g-C_3_N_4_/CB material for the detection of heavy metal ions. The detection sensitivity of the g-C_3_N_4_/CB electrochemical sensor under visible light irradiation is greatly improved under dark conditions (Figure 5c). Stripping voltammetry includes two processes: enrichment and dissolution. By applying an applied voltage, the target pollutant in the solution is enriched on the electrode surface, and a reverse voltage is applied to dissolve the metal or insoluble film enriched on the electrode surface [82]. The g-C_3_N_4_ material is excited under visible light irradiation to generate electrons (e^−^) and holes (h^+^) in the conduction band and valence band, respectively. Photogenerated electrons act as a reducing agent to promote the reduction of heavy metal ions to the metal state on the electrode surface. In the electrochemical dissolution process, photogenerated holes are beneficial to the oxidation of heavy metals into corresponding ions (Figure 5d).

In summary, the intrinsic properties of g-C_3_N_4_ and the potential electrochemical detection mechanisms indicate that g-C_3_N_4_ nanomaterials are promising candidates for constructing electrochemical sensors toward heavy metal ions. Especially, its tunable surface chemistry, unique electronic structure, and high stability ensure its environmental monitoring application.

## 4. Application of g-C_3_N_4_ in Heavy Metal Detection

Bulk g-C_3_N_4_ has been considered to have a similar layered structure to graphite; with its unique electronic energy band structure, high thermochemical stability, and low-cost precursor materials, g-C_3_N_4_ has attracted people’s attention in the photocatalytic degradation of environmental organic pollutants and electrochemical sensor detection of heavy metal ions [83,84]. However, the high recombination rate of photoexcited charge carriers and the small specific surface area in the bulk g-C_3_N_4_ system led to low photocatalytic activity [85,86]. Therefore, many approaches have been used to address these limitations, such as changing the morphology of g-C_3_N_4_ or a composite with other nanoparticles as a template. Mainly, the excellent adsorption and catalytic properties of g-C_3_N_4_ nanomaterials brought new insights to utilize them as an electrochemical modifier for heavy metal ion detection. In the following sub-sections, recent advances in g-C_3_N_4_ and g-C_3_N_4_-based nanocomposites for heavy metal ion sensing are discussed, and their sensing performances are presented in Table 1.

### 4.1. g-C_3_N_4_ Nanofibers/Tubes

**Figure 6:**
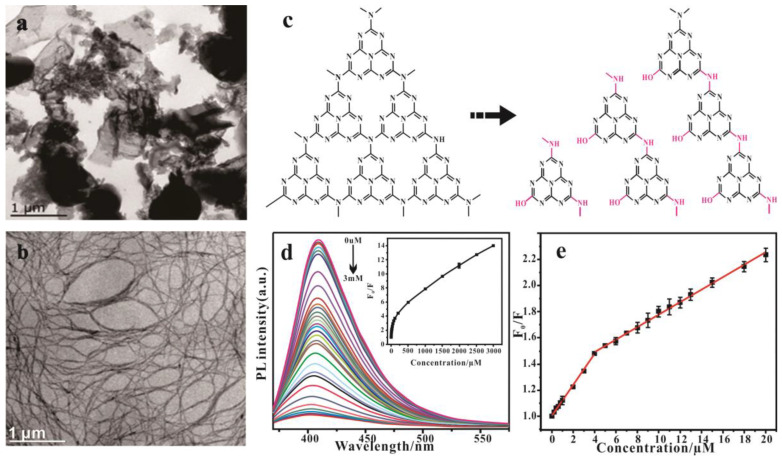
(**a**) Proposed preparation mechanism of g-C_3_N_4_ nanofibers; (**b**) TEM image of bulk g-C_3_N_4_; (**c**) g-C_3_N_4_ nanofibers; (**d**) fluorescence quenching of g-C_3_N_4_ nanofibers in the presence of different DA concentrations. The inset figure presents the relationship between F_0_/F and the concentration of DA. (**e**) The corresponding linear relationship between F_0_/F and the concentration of DA [118].

Low-dimensional functional nanomaterials have attracted much attention due to their high specific surface area and excellent electronic and optical properties [112,113]. Compared with bulk g-C_3_N_4_, one-dimensional structures such as g-C_3_N_4_ tubes or rods have a higher aspect ratio, which can guide the electrons to move along the axial direction and limit the lateral transfer of electrons, thereby inhibiting photo-generated carrier recombination [114,115]. At the same time, this structure usually has a larger specific surface area, so that its surface has more reactive active sites, which is conducive to the improvement of catalytic reaction performance [116]. In addition, the highly ordered array of nanotube rod structures can provide an efficient electron migration path for electron-hole transport. The one-dimensional structure of g-C_3_N_4_ is synthesized using chitosan as a template [117]. Lv et al. [118] successfully prepared g-C_3_N_4_ nanofibers by hydrolysis under alkaline conditions. Figure 6a is the proposed preparation mechanism of g-C_3_N_4_ nanofibers; the g-C_3_N_4_ nanofibers show an ultrathin structure and are up to a micron in length (Figure 6c). Compared with bulk g-C_3_N_4_, the prepared g-C_3_N_4_ nanofibers have higher fluorescence quantum yield and larger specific surface area. In addition, g-C_3_N_4_ nanofiber fluorescent probes have achieved high selectivity and sensitivity for DA detection, with a minimum detection limit of 17 nM (Figure 6d,e). Tian et al. [119] quickly prepared ultrathin C_3_N_4_ nanofibers with a diameter of 5–10 nm from top to bottom through alkaline catalytic hydrolysis. The morphology of g-C_3_N_4_ nanostructures can be controlled by reaction time. A fluorescence sensor was constructed using the prepared ultrathin C_3_N_4_, which can detect Fe^3+^ rapidly and selectively. Zheng et al. [120] synthesized helical g-C_3_N_4_ rods by nanometer casting using mesoporous silica as templates. Compared with bulk g-C_3_N_4_, the spiral structure of g-C_3_N_4_ promotes the charge separation and mass transfer of carbonitride semiconductors, thus significantly improving the catalytic efficiency of water decomposition and carbon dioxide reduction. Dimitra et al. [121] synthesized rod graphitic carbon nitride (RGCN) by a chemical method, which showed obvious selectivity to Cu^2+^ ions. The influence of morphology on the Cu^2+^ sensor was further investigated, and the thin sheet-like graphitic carbon nitride (SGCN) was synthesized. Compared with SGCN, RGCN shows a stronger Cu^2+^ sensing capability due to its porous rod structure and high nitrogen content. Wang et al. [95] reported a highly sensitive electrochemical sensor based on a sulfur-doped C_3_N_4_ bundle with hierarchical pores/graphene composites (STB/Gs-x) for the detection of trace heavy metal ions in the environment. The sulfur-containing GCN tube bundle (STB) was constructed by a one-step method. The focus is on the synergistic effect of the layered porous tube bundle structure and S atom doping on the detection of HMIs, which further solves the deficiencies of b-g-C_3_N_4_ in the detection of electrochemical HMIs.

### 4.2. g-C_3_N_4_ Nanosheets

In recent years, inspired by the methods for preparing graphene and graphene-like materials, the bulk g-C_3_N_4_ can be exfoliated into a single or few layers of graphene-like C_3_N_4_ [122,123]. The thickness of nanosheets is generally at the nanometer or sub-nanometer level, and due to their unique two-dimensional anisotropic structural characteristics and quantum-filling effects, nanosheets have new physical and chemical properties [124,125]. It was found that 2D layered materials showed superior electrochemical performance in detecting heavy metal ions compared with 3D block materials [126]. The bulk C_3_N_4_ has a stacked layered structure in which each layer is composed of C–N covalent bonds with strong bonding force, but the bond energy between the layers is the van der Waals force with weak bond energy. Therefore, it is feasible to obtain C_3_N_4_ nanosheets by bulk C_3_N_4_ [127]. Nanosheets have the following advantages: (1) Having a large specific surface area can provide rich reactive sites. (2) With a short bulk diffusion length, the recombination of photo-excited charge carriers can be reduced. (3) Has a large band gap, which helps to enhance the redox capacity of charge carriers. (4) The photophysical behavior of the photo-excited carriers has changed compared with the bulk C_3_N_4_, which can extend the life of the charge carriers.

The preparation methods of g-C_3_N_4_ nanosheets can be divided into “top-down” and “bottom-up” [128]. The ultrasonic liquid exfoliation method and the secondary thermal oxidation etching method are the most commonly used to prepare g-C_3_N_4_ nanosheets from the “top-down” methods. In each of these methods, it is synthesized from a block structure by a two-step process. Bulk C_3_N_4_ was prepared by thermal polymerization and then transformed into nanosheets by exfoliating and etching. This approach offers advantages such as simple operation, low cost, and the ability to retain the inherent structure of g-C_3_N_4_. However, it faces challenges in precisely controlling the thickness and size distribution of the nanosheets during exfoliation. Additionally, the exfoliated nanosheets may restack, reducing the exposure of active sites. In contrast, “bottom-up” methods directly synthesize nanosheet structures through the condensation polymerization of molecular precursors (e.g., melamine and urea), including techniques such as hard-template, soft-template, and molecular self-assembly approaches. These methods provide advantages such as controllable structures, high specific surface area, and fewer defects. Nevertheless, they suffer from limitations like complex procedures and low yield.

**Figure 7:**
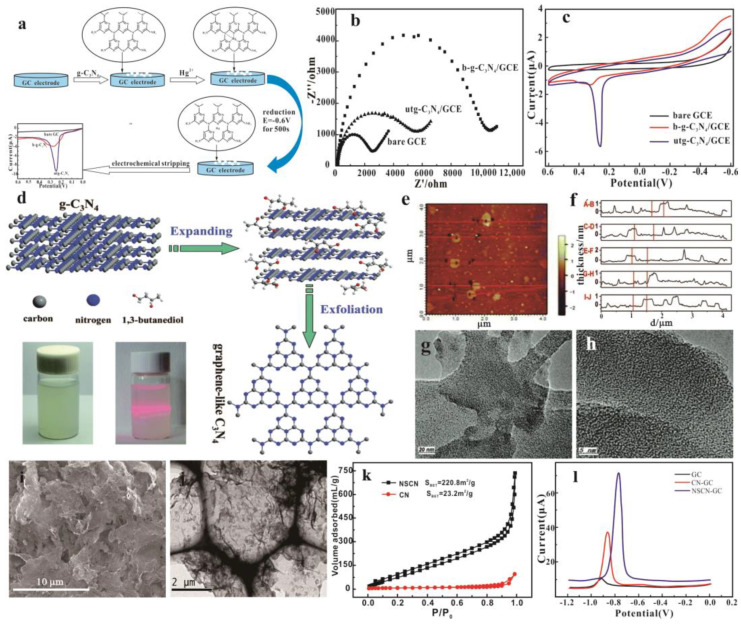
(**a**) Illustration of the electrochemical sensing of Hg^2+^ ions using a utg-C_3_N_4_/GC electrode. Electrochemical impedance spectra (**b**) and cyclic voltammograms (**c**) of bare GC, bg-C_3_N_4_/GC, and utg-C_3_N_4_/GC electrodes [130]. (**d**) Schematic illustration of liquid-exfoliation process from the bulk g-C_3_N_4_ to graphene−like C_3_N_4_. (**e**) AFM image of the prepared graphene-like C_3_N_4_. (**f**) The corresponding height image of the five randomly chosen graphene-like C_3_N_4_ sections. (**g**) TEM image of the graphene-like C_3_N_4_. (**h**) HRTEM image of the graphene-like C_3_N_4_ [132]. SEM and TEM images of NSCN (**i**,**j**). (**k**) N_2_ adsorption–desorption isotherms of CN and NSCN. (**l**) The detection of Cd^2+^ on bare GC, CN-GC, and NSCN-GC (the concentration of Cd^2+^ is 10 μM) [72].

Graphite carbon nitride has undergone sufficient protonation and ultrasonic treatment to synthesize g-C_3_N_4_ nanosheets. Its properties are similar to two-dimensional films, with enhanced ion conductivity and a large specific surface area. Density functional theory (DFT) calculations show that the high level of protonation makes g-C_3_N_4_ have better conductivity [129]. Hatamie et al. [90] prepared a large number of bulk g-C_3_N_4_ via high-temperature polymerization of melamine and obtained g-C_3_N_4_ nanometer tablets by sonication-assisted liquid exfoliation. Using g-C_3_N_4_ nanosheets to modify glassy carbon electrodes can achieve trace detection of lead ions in a water environment; the detection limit is 1 ng·L^−1^. Using the same method, Zhang et al. [130] prepared ultrathin g-C_3_N_4_ (Utg-C_3_N_4_) nanosheets with a thickness of about 8 mm. Compared with g-C_3_N_4_, the Utg-C_3_N_4_-modified GC electrode has an enhanced electrochemical response to Hg^2+^, which could be ascribed to the strong affinity between utg-C_3_N_4_ and Hg^2+^ through its single bond –NH and single bond –NH_2_ groups (Figure 7a). This allows for the detection of Hg^2+^ in aqueous solutions with high sensitivity and selectivity (Figure 7b,c). In addition, studies have shown that graphene-like carbonitrides can be synthesized by liquid phase exfoliation in 1,3-butanediol (Figure 7d). The obtained graphene-like C_3_N_4_ has a two-dimensional thin-layer structure with a thickness of about 3–6 atoms and a specific surface area of 32.54 m^2^g^−1^ (Figure 7e–h). The photocurrent response, electron transfer ability, and photocatalytic activity are all enhanced. In another work, Liu et al. [24] successfully prepared the activated ultrathin g-C_3_N_4_ nanosheets by bulk graphitic carbon nitride (g-C_3_N_4_) to liquid ultrasonic exfoliation and protonation treatment. An electrochemical sensing interface was developed using activated g-C_3_N_4_ nanosheets modified with a glassy carbon electrode for the determination of Cd^2+^, with the obtained sensitivity and detection limit being 22.668 µA/µM and 3.9 nM, respectively. In addition, high spiked recoveries were obtained in the detection of Cd^2+^ in natural water and rice samples using the prepared a g-C_3_N_4_ modified electrode. This result can be attributed to the ultrathin layer and high surface for the effective accumulation of metal ions required for enhanced sensitivity.

The liquid phase stripping method has the advantages of simple and direct operation, but its yield is very low, and it is time-consuming. Thus, a new synthesis idea was developed: the precursors of g-C_3_N_4_ were pretreated and thermally polymerized from bottom to top to directly synthesize nano-flake g-C_3_N_4_. Sadhukhan et al. [131] synthesized two-dimensional carbon nitride by a microwave-assisted method. The g-C_3_N_4_ nanosheets were formed by evaporation-induced self-assembly of nitride carbon points on the fixed substrate by microwave radiation of formamide. In addition, a g-C_3_N_4_ nanosheet-modified glassy carbon electrode can be used for the highly sensitive detection of mercury ions in an aqueous solution, with a minimum detection limit of 9.1 × 10^−11^ M. Using melamine as a raw material and acetic acid as a bubble template, Wang et al. [72] prepared graphitized carbon nitride ultrathin nanosheets by thermal polymerization taking melamine as raw materials and deionized water and acetic acid as bubble templates. During the thermal polymerization process, acetic acid decomposes to produce a large number of bubbles, which is beneficial to the formation of CN nanosheets (Figure 7i,j). BET data (Figure 7k) show that the specific surface area of ultrathin nanosheets is 10 times that of g-C_3_N_4_, which indicates that there are more active sites to adsorb Cd^2+^. Then, an electrochemical-sensitive interface based on a g-C_3_N_4_ nanosheet was constructed for the detection and analysis of cadmium ions in an aqueous environment (Figure 7l). The results showed that the electrochemical sensor had a low detection limit (0.35nm) and had potential application potential in the detection of heavy metals. Zou et al. [91] reported that the sulfur-doped carbon nitride nanosheet-modified glassy carbon electrode (S-g-C_3_N_4_/GCE) has the best large specific surface area and good lead ion (Pb^2+^) detection ability. The results of DFT and XPS show that S doping mainly replaces the N atoms in the g-C_3_N_4_ molecular framework.

### 4.3. The Mesopores g-C_3_N_4_

The attractive properties of mesoporous materials, including a high specific surface area and a controllable mesoporous structure, make them attractive subjects for many applications. Recent developments in mesoporous materials have improved the performance of these sensors by the immobilization of receptors to large and accessible surface areas and well-defined pores, which facilitate a high adsorption capacity for chromogenic/fluorescent molecules and the effective transport of analytes and hence detection limits below tens of nanomolar concentrations. In 2005, mesoporous carbon nitrides with different pore diameters were synthesized for the first time [133]. Compared with bulk g-C_3_N_4_, it significantly increased the specific surface area while maintaining the same physical and chemical properties, thereby improving its application in catalysis and adsorption. On the one hand, the presence of mesopores increases the specific surface area of g-C_3_N_4_ and provides more active reaction sites for metal ions. On the other hand, the hollow structure facilitates the transfer of electric charges and improves the sensitivity of electrochemical reactions.

**Figure 8:**
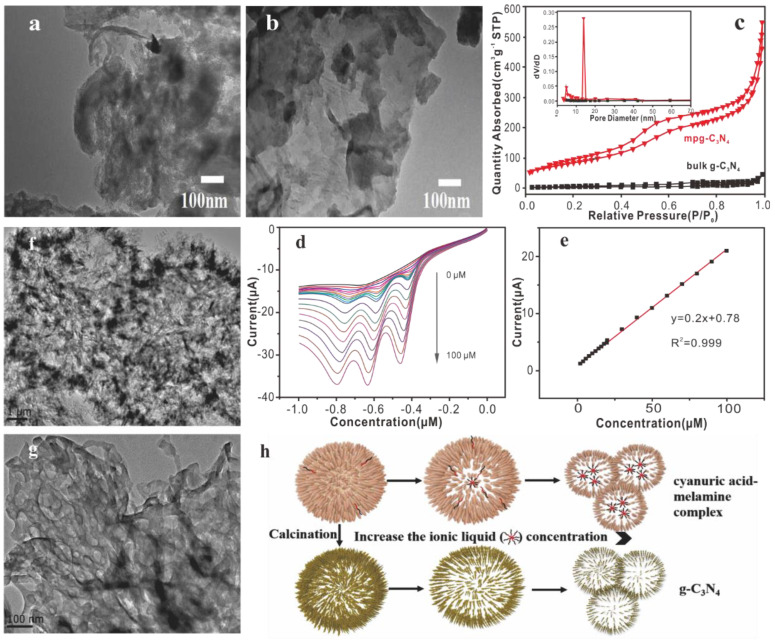
mpg-C_3_N_4_ (**a**) and mpg-C_3_N_4_/β-CD (**b**). (**c**) The N_2_ adsorption−desorption isotherms and the corresponding pore size distribution curves (inset) of bulk g-C_3_N_4_ and mpg-C_3_N_4_. (**d**) mpg-C_3_N_4_/β-CD-modified GCE at different concentrations with the response to the blank solution in 0.5 M PBS (pH 7.0). (**e**) Calibration plot of the peak currents vs. the corresponding TNT concentrations [139]. (**f**,**g**) TEM images of CN-E_0.08_. (**h**) Formation mechanism of hollow mesoporous carbon nitride spheres [140].

The template method is one of the most effective methods to prepare MCN with controllable pore size, morphology, and structure. It is mainly divided into the hard template method and the soft template method. The hard template method is used to synthesize MCN mainly by immersing the precursor in the silica template such as SBA-15 [134], FDU-12 [135], KIT-6 [136], etc., polymerizing and crosslinking the carbon-containing precursor and the nitrogen-containing precursor at high temperature to form mesoporous CN material, and then cleaning with HF to remove the silica template [137]. The soft template method is a method in which surfactants, amphiphilic block polymers, or ionic liquids are used as templates for synthesis. The soft-template synthesis is mainly through the formation of different emulsions to obtain the micellar phase used as a template. The g-C_3_N_4_ precursor undergoes condensation around the soft template, and the soft template is removed by calcination polymerization [138]. Wang et al. [139]. prepared mesoporous graphite-carbonitride (mpg-C_3_N_4_) by the hard template method and proposed the use of mpg-C_3_N_4_ and β-cyclodextrin (β-CD) nanocomposites to modify glassy carbon working electrodes for the detection of trace amounts of TNT. It can be observed from the TEM image that mpg-C_3_N_4_ has a stacked lamellar structure and tiny mesopores (Figure 8a,b). BET measurement results show that g-C_3_N_4_ does not have obvious pores. For mpg-C_3_N_4_ samples, the pores are mainly distributed in the range of 3–20 nm (Figure 8c). The sensitivity of mpg-C_3_N_4_/β-CD nanocomposite for detecting TNT is 0.2 μA/μM, and the detection limit is 68ppb (Figure 8d,e). The mpg-C_3_N_4_/β-CD nanocomposite is used to capture nitroaromatic hydrocarbons near the electrode–solution interface to promote electrochemical reduction. Zhao et al. [140]. used a one-step soft template method to synthesize hollow mesoporous g-C_3_N_4_ spheres with high specific surface area and high porosity. It utilizes the supramolecular assembly of cyanuric acid and melamine through hydrogen bonds and the structure-oriented properties of ionic liquids. And the corresponding formation mechanism is placed in Figure 8h. It can be seen from Figure 8f,g that there are a large number of mesopores in the structure of CN, which may be caused by ionic liquid micelles, NH_3,_ and CO_2_ gases. In addition, the porous structure and ultrathin nanosheets facilitate the exposure of active sites, thereby increasing the interface charge transfer rate.

According to previous reports, the pore structure of these mesoporous materials is conducive to enhancing the light-harvesting capacity and the ability to adsorb pollutants and significantly improving the photocatalytic activity. Therefore, the preparation of mesoporous g-C_3_N_4_ may be a promising method to solve the defects of the g-C_3_N_4_ body.

### 4.4. g-C_3_N_4_-Based Nanocomposites

#### 4.4.1. Metal Nanoparticles/g-C_3_N_4_ Nanocomposites

Metal nanoparticles such as gold nanoparticles (AuNPs) and silver nanoparticles (AgNPs) have the characteristics of small size and a large specific surface area. At the same time, their unique optical, electronic, magnetic, and antibacterial properties have also received extensive attention [141]. The precursors and synthesis methods of metal nanoparticles play an important role in their performance. In general, metal salts are used as precursors to be reduced to the corresponding metal nanoparticles. The stabilizer is attached to the surface of the metal nanoparticles, effectively inhibiting the agglomeration of the metal nanoparticles. The metal nanoparticle-modified electrode can control the functionalization of the required groups and increase the mass transfer rate and specific surface area, thereby enhancing the dynamic performance of the electrode.

**Figure 9:**
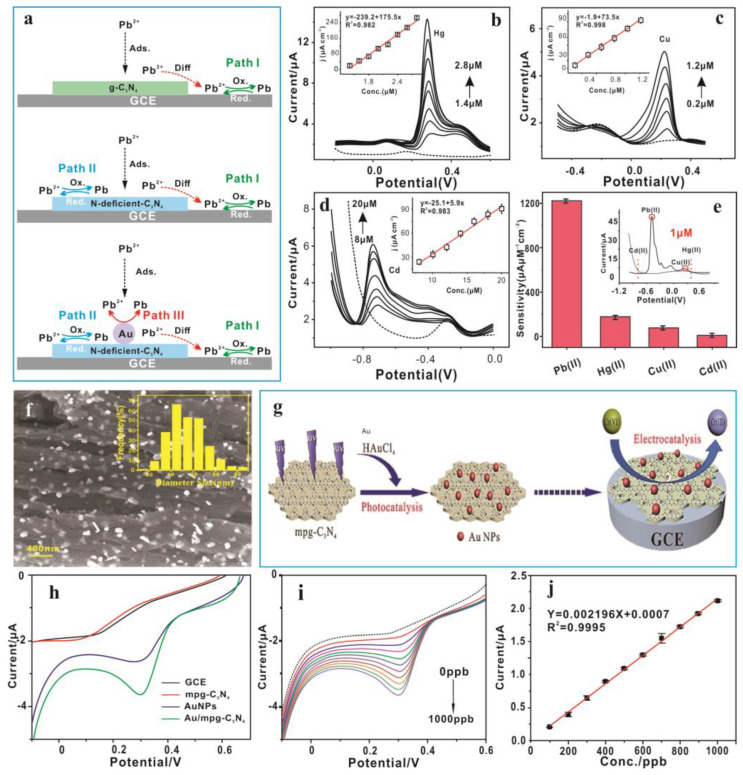
SWASV responses and linear equations (inset) for the detection of (**a**) Pb(II); (**b**) Hg(II); (**c**) Cu(II); and (**d**) Cd(II) with Au/N-deficient-C_3_N_4_/GCE (Dashed line: blank). (**e**) Comparisons of the sensitivity of the three modified electrodes. (**f**) Synergistic catalysis mechanism toward Pb(II) based on three different nanomaterials modified with GCE. (**g**) Schematic illustration of the synthesis of the Au/mpg-C_3_N_4_ used to construct electrochemical sensors for Cr(VI). (**h**) The LSV responses for 1000 ppb Cr(VI) with modified GCE at a scan rate of 100 mV/s in 0.1 M HCl. (**i**) Variation in response current as a function of Cr(VI) concentration as determined by LSV. (**j**) Calibration curve of Cr(VI) vs. response current [7].

The g-C_3_N_4_ nanocomposite materials based on metal nanoparticles are considered to be effective, strong, and stable electrochemical sensing materials. Among them, AuNPs/g-C_3_N_4_ nanocomposites are the most widely used electrochemical modification materials. Xiao et al. [99] found that in Au/N-deficient-C_3_N_4_, N vacancy defects and Au nanoparticles have a synergistic catalytic effect, which can be used for high-sensitivity electrochemical detection of Pb(II) and has anti-interference properties. Figure 9a–d show the SWASV responses for the detection of Pb(II), Hg(II), Cu(II), and Cd(II). It can be concluded that the sensitivity of Au/N-deficient-C_3_N_4_/GCE towards Pb(II) is the best (Figure 9e). Figure 9f describes the electrocatalytic mechanism of Pb(II) on the sensitive interface modified by three different nanomaterials and clearly illustrates the significance of the synergistic catalytic effect of N vacancies and ~5 nm Au nanoparticles. The N vacancies in g-C_3_N_4_ could greatly improve the selective adsorption of Pb(II), and ∼5 nm Au nanoparticles enhanced the catalysis of materials. Chen et al. [93] developed a green method to synthesize AuNPs/mpg-C_3_N_4_ nanocomposites for constructing an anti-interference electrochemical sensing interface toward methylmercury, which might be used in the field of the fast screen of methylmercury in food and the environment. In one of their works, the electrochemical sensor constructed as an electrode modification material can directly, quickly, and accurately analyze Cr(VI) in wastewater samples from factories such as leather wastewater and electroplating wastewater [7]. The electrochemical test results yielded 376 ppb of Cr(VI) in TW, which is similar to the ICP-OES result (386 ppb) with a recovery of 97.4%. It is shown that the constructed electrochemical sensor is suitable for the analysis of real water samples in complex situations and has a strong potential for practical application. Mesoporous graphite phase carbon nitride and chloroauric acid were used as photocatalysts and raw materials, respectively, and successfully reduced gold nanoparticles to the surface and interlaminar gaps of mesoporous graphite phase carbon nitride under the irradiation of ultraviolet light (Figure 9g). Figure 9h shows the LSV response of different material modifications to Cr(VI). The peak current of Au/mpg-C_3_N_4_/GCE is almost double that of AuNPs/GCE. As shown in Figure 9i,j, a calibration curve in the range 100~1000 ppb is developed using an Au/mpg-C_3_N_4_ sensor by the electrochemical method, with the obtained sensitivity and detection limit of Cr(VI) being 0.002196 µA/ppb and 14.7 ppb, respectively. The excellent performance is probably due to the charge transfer between gold nanoparticles and g-C_3_N_4_, which induces a synergistic catalytic effect. The optimal UT-g-C_3_N_4_/Ag hybrids displayed a faster electron transfer rate in comparison to UT-g-C_3_N_4_ and bulk g-C_3_N_4_. The preparation of Pt/g-C_3_N_4_/polythiophene nanocomposites (Pt/g-C_3_N_4_/PTh) as an electrochemically sensitive material for the determination of Hg^2+^ is presented by Mahmoudian et al. [89]. The results showed an increase in the active sites due to the presence of the g-C_3_N_4_ which significantly enhanced the absorption of Hg^2+^. The presence of PTh and PtNPs compensated for the lower electrical conductivity of the g-C_3_N_4_ in the nanocomposite. Nanometer zero-valent iron (nZVI) is one of the most widely used nanomaterials for groundwater and hazardous waste treatment due to its high reduction rate and removal capacity [142]. g-C_3_N_4_ can effectively disperse nZVI while adjusting its microstructure, further achieving efficient adsorption and reduction of target pollutants. The results show that G-nZVI has a better affinity for Pb(II) than naked nZVI, mainly depending on the change in microstructure to improve the adsorption capacity of heavy metal ions and synergistically enhance the reaction activity and stability [143]. Meanwhile, the high catalytic capacity of metal nanoparticles is a key factor in increasing the electrochemical performance of metal nanoparticles/g-C_3_N_4_ nanocomposites. There will be good prospects in the future for metal nanoparticles/g-C_3_N_4_ nanocomposites in electrochemical redox reactions.

#### 4.4.2. Metal Oxide Nanomaterials/g-C_3_N_4_ Nanocomposites

Metal oxide nanomaterials have low environmental toxicity and strong biocompatibility. Due to their strong adsorption capacity, larger specific surface area, and better electron transfer kinetics, they have good physical and chemical properties and high-efficiency catalysis. Their original form and combination with other materials have excellent electrochemical properties and are suitable for use as electrode modification materials to achieve the purpose of capturing target pollutants in water with high selectivity. Among the electrode modification materials currently used for electrochemical analysis of heavy metal pollutants, MnO_2_ [144], ZnO [145], CuO [146], Fe_3_O_4_ [147], ZrO_2_ [148], TiO_2_ [149], etc., are the most commonly used nano metal oxide materials.

**Figure 10:**
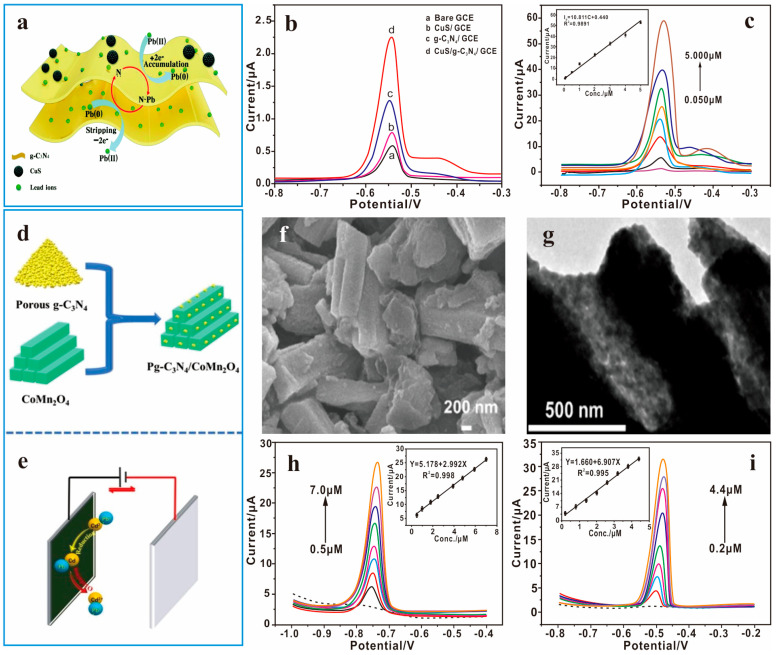
(**a**) Mechanism of the proposed sensor for Pb^2+^ detection based on the CuS/g-C_3_N_4_ electrode. (**b**) SWASV responses of 0.2 μM Pb^2+^ at four different working electrodes in the NaAc/HAc buffer solution. (**c**) SWASV detection of Pb^2+^ in NaAc/HAc buffer solution with the CuS/g-C_3_N_4_/GCE [101]. (**d**) Schematic illustration of the synthesis process of pg-C_3_N_4_/CoMn_2_O_4_ nanocomposite. (**e**) The reduction mechanism of Cd(II) at the electrochemical sensitive interface. SEM images (**f**) and TEM images (**g**) of pg-C_3_N_4_/CoMn_2_O_4_ nanocomposite; (**h**) SWASV responses at pg-C_3_N_4_/CoMn_2_O_4_/GCE in 0.1 M HAc-NaAc containing various concentrations of Cd(II). (**i**) SWASV responses at pg-C_3_N_4_/CoMn_2_O_4_/GCE in a 0.1 M HAc–NaAc containing various concentrations of Pb(II) (Dashed line: blank).

The incorporation of metal oxide materials increases the oxygen vacancies and defects of the composite material, creating key active sites for the catalytic reaction [150]. Wang et al. [151] present a novel method to prepare mesoporous materials via in situ self-assembly of graphitic carbon nitride nanosheets and SiO_2_ nanoparticles. Combining the advantages of g-C_3_N_4_ nanosheets and a mesoporous structure, the as-prepared materials exhibit superior adsorption capabilities for heavy metal ions and organic pollutants. The band gap of g-C_3_N_4_ can be reduced by doping CuO, and the electron transfer resistance of the interface can be enhanced. At the same time, the strong coordination mode between the N and Cu atoms at the interface of the CuO-g-C_3_N_4_ framework heterojunction is beneficial to improving the electrochemical performance [146]. The CuO/g-C_3_N_4_ material prepared by Atacan has remarkable repeatability and good stability for H_2_O_2_ electrochemical sensors [152]. As a common semiconductor material, copper sulfide (CuS) can be used as a functional component to improve the electrical properties of matrix materials because its conductivity is comparable to that of metals. Ning et al. [101] successfully prepared a new type of electrochemical sensor based on CuS/g-C_3_N_4_/GCE, which can effectively amplify the SWASV signal of Pb^2+^ (Figure 10b,c). The limit of detection (LOD) for Pb^2+^ is calculated to be 4.00 nM (S/N = 3). Figure 10a shows the possible reduction mechanism of Pb^2+^ at the CuS/g-C_3_N_4_ interface. The higher response of Pb^2+^ may be due to the outstanding advantages of this multifunctional composite material: (i) the complementary effect of “dispersibility/electrical conductivity”. (ii) A synergistic enrichment effect of “coordination/adsorption” [101]. In addition, binary transition metal oxides have better catalytic activity and conductivity than single metal oxides. Wang et al. [105] integrated pg-C_3_N_4_ and CoMn_2_O_4_ into nanocomposite materials and developed a heavy metal ion detection probe with high sensitivity and selectivity (Figure 10d,e). It was observed by SEM and TEM that pg-C_3_N_4_ was wrapped on the surface of CoMn_2_O_4_ (Figure 10f,g). The pg-C_3_N_4_/CoMn_2_O_4_ nanocomposite has abundant accessible active sites and a high-efficiency electron transport path, which can realize the selective capture of Cd(II) and Pb(II) (Figure 10h,i). Furthermore, the precise coupling of g-C_3_N_4_ with CuCo_2_O_4_ can enhance the performance for Hg^2+^ reduction through the heterostructure formation [153]. Li et al. [154] combined low-pressure ultraviolet (LPUV) photolysis with a ZnO/g-C_3_N_4_ photocatalyst to enhance the SWASV signal of heavy metal ions (HMIs). In the analysis of HMIs in real soil extracts, the sensing system achieved an accuracy of 94.9% and 99.8% for the detection of Cd^2+^ and Pb^2+^, respectively, which verified the feasibility and validity of the proposed method for environmental applications.

Metal oxide nanoparticle/g-C_3_N_4_ nanocomposites have emerged as high-performance materials for the electrochemical detection of heavy metal ions, leveraging the synergistic interplay between g-C_3_N_4_’s rich surface chemistry and the catalytic/conductive properties of metal oxides. Among them, metal oxides can enhance redox activity and electron transfer kinetics, enabling ultrasensitive detection via stripping voltammetry [155]. Functional oxides can improve selective adsorption of target ions through electrostatic interactions, ion exchange, or surface complexation. Magnetic oxides enable easy electrode regeneration via external magnetic fields, addressing fouling issues and extending sensor lifespan.

#### 4.4.3. Carbonaceous Nanomaterials/g-C_3_N_4_ Nanocomposites

Carbon-based nanomaterials have attracted widespread attention in the field of electrochemical sensors due to their unique properties. Carbon-based nanostructured materials include fullerenes, carbon nanotubes (CNTs), graphene (G) and its derivatives (graphene oxide (GO)), nano-diamonds (NDs), and carbon-based quantum dots (CQDs). Carbon nanotubes (CNTs) have high tensile strength and strong hardness, and due to their one-dimensional nanotube structure, their electrical and thermal conductivity have been significantly improved, even surpassing some conductive metals [156]. These excellent properties have important significance for improving the sensitivity of electrochemical sensors.

**Figure 11:**
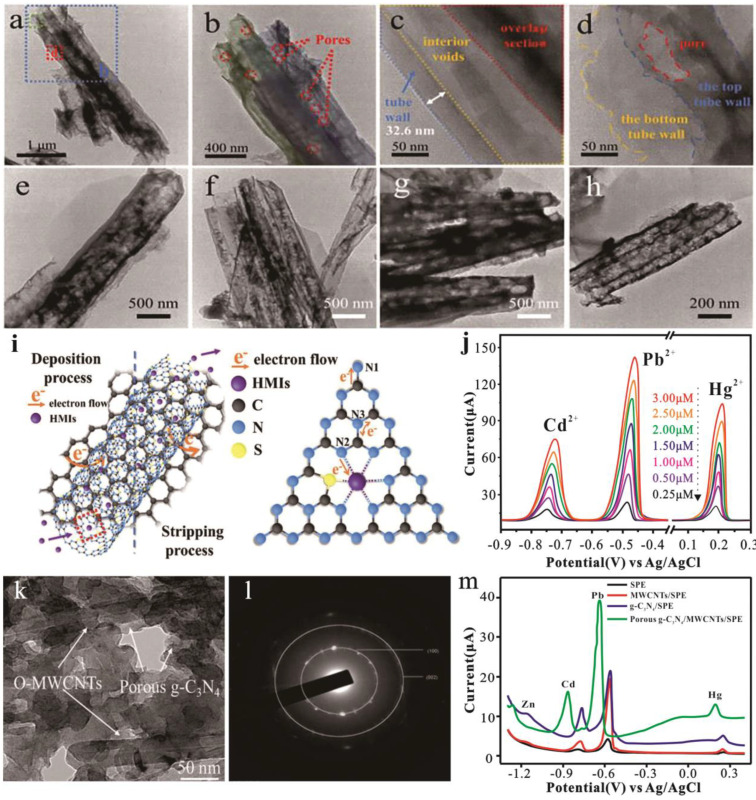
TEM images of (**a**–**d**) STB and (**e**–**h**) STB/Gs-2. (**i**) Schematic diagram of SWASV process and electron transfer of STB-adsorbed HMIs. (**j**) Simultaneous detection SWASV curves of STB/Gs-2 modified electrode. (**k**) HR-TEM images of porous g-C_3_N_4_/O-MWCNTs; (**l**) SAED pattern of porous g-C_3_N_4_/O-MWCNTs. (**m**) DPV response of different modified electrodes in 0.1 M acetate buffer (pH −5.0) containing 0.5 μg L^−1^ of Hg and 25 μg L^−1^ of Pb, Cd, Zn metals.

In the research conducted by Wang et al. [95], the synergistic effect between sulfur-doped C_3_N_4_ tube bundles (STBs) with hierarchical pores and graphene nanosheets (Gs) is beneficial for ultra-trace heavy metal ion (HMI) detection and makes the STB/Gs composite a promising sensitive electrochemical sensor for HMIs detection. It can be seen from the TEM image (Figure 11 a–d) that there are many small holes in the tube wall, and these pores promote the rapid diffusion of the HMI solution in the tube bundle structure. The STB/Gs-2 composite is shown in Figure 11e–h. The STB could be seen scattered on the surface of Gs. When STB active sites adsorbed HMIs, Gs could rapidly transfer electrons between STB active sites and electrodes to realize the rapid electrochemical REDOX reaction of HMIs deposition and stripping processes [95]. The SWASV curves of the STB/Gs-2-modified electrode were demonstrated in Figure 11j. The STB/Gs-2-modified electrodes showed that the LOD was 1.17, 0.38, and 0.61 nM for simultaneous detection and 2.30, 0.78, and 1.15 nM for individual detection of Cd^2+^, Pb^2+^, and Hg^2+^. Thin g-C_3_N_4_ nanosheets were prepared by acid–base corrosion and an ultrasonic-assisted method, using g-C_3_N_4_ NS/RGO to construct an electrochemical sensing interface for the detection of Pb^2+^ and Cd^2+^ [157]. The reduced graphene oxide sheet acts as a conductive channel to promote the electron–hole separation and transfer rate in g-C_3_N_4_ NS, thereby improving the photocurrent intensity and photocatalytic performance. Studies have shown that the composite of g-C_3_N_4_ and CB can create a new synergistic effect while maintaining the characteristics of each material, which can promote the effective detection of toxic heavy metals in the solution [81]. Under visible light conditions, the detection sensitivity of the g-C_3_N_4_/CB electrochemical sensor is greatly improved than under dark conditions. The photo-assisted electrochemical sensor has good detection stability and reproducibility and can detect multiple different heavy metal ions at the same time, and the detection limit (LOD) is very low. The performance of carbon nanotubes largely depends on the way they are rolled. Among them, multi-walled carbon nanotubes (MWCNTs) have more excellent properties. In the research of Ramalingam et al. [107], three-dimensional nanocomposites composed of porous graphitic carbon nitride nanosheets (p-g-C_3_N_4_-NSs) and oxidized multiwalled carbon nanotubes (O-MWCNTs) were prepared by chemical oxidation (Figure 11k,l). One-step oxidation has two main effects. One is to cause the formation of acidic functional groups on both the basal plane of g-C_3_N_4_ and MWCNTs, and the other is that O-MWCNTs bind in situ to the porous structure of p-g-C_3_N_4_. In Figure 11m, the nanocomposite material used to modify the screen-printing electrode (SPE) to have good sensitivity and selectivity to heavy metal ions Cd(II), Hg(II), Pb(II), and Zn(II) can be seen. Recently, MOFs have been used as electrode materials to detect HMIs on account of their excellent ability to bind heavy metal ions, high specific surface area, and easy modification. Chen et al. combined the advantages of g-C_3_N_4_ and ZIF-8 and developed a g-C_3_N_4_@ZIF-8 nanocomposite material, which is suitable as a sensitive material for detecting metal ions and solvents [158]. Chen et al. [97] modified GCE with g-C_3_N_4_/CNT/NH_2_-MIL-88(Fe) as a modifier, and used square wave stripping voltammetry to simultaneously detect Cd^2+^, Pb^2+^, Cu^2+^, and Hg^2+^. The doping of g-C_3_N_4_ in the composite, rich in N-containing functional groups, participates in the adsorption of metal ions on the surface of the electrodes. The porous composite provides accommodation room for metals generated by electro-reduction. The detection limit for Cd^2+^, Pb^2+^, Cu^2+,^ and Hg^2+^ is 39.6 nM, 7.6 nM, 11.9 nM, and 9.6 nM, respectively. In addition, Ti_3_C_2_T_x_ and g-C_3_N_4_ are potential complementary materials for the preparation of stable and conductive composite materials, which exhibit excellent electrochemical performance. The protonated g-C_3_N_4_/Ti_3_C_2_T_x_ electrode exhibited remarkable sensitivities for the simultaneous detection of Cd^2+^ (0.05∼1.50 μM) and Pb^2+^ (0.05∼1.50 μM), and the detection limits of Cd^2+^ and Pb^2+^ were 0.001 and 0.0006 μM, respectively [108].

In summary, carbon-based materials enhance the electrochemical performance of g-C_3_N_4_ by addressing its inherent limitations, including low electrical conductivity and limited active site accessibility. Key advancements include the following: (1) enhanced conductivity: integration with graphene or CNTs creates conductive networks that accelerate electron transfer kinetics, improving sensitivity in techniques; (2) active site engineering: functionalized carbon materials (e.g., oxygen/nitrogen-doped graphene) coupled with g-C_3_N_4_ increase adsorption capacity and catalytic activity for target ions via π–π interactions, electrostatic binding, or chelation. (3) Stability: carbon coatings (e.g., graphitic shells) protect g-C_3_N_4_ from oxidative degradation in harsh environments, ensuring long-term operational reliability.

#### 4.4.4. Conductive Polymer/g-C_3_N_4_ Nanocomposites

**Figure 12:**
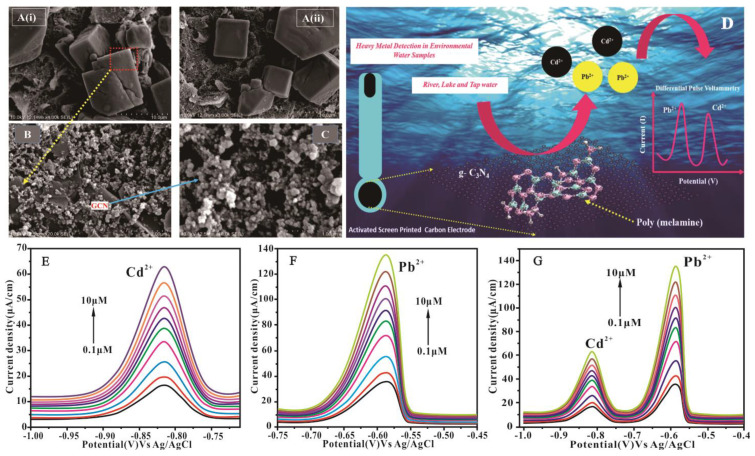
Structural morphology of (**A**) (**i**) and (**ii**) ASPE-PM microcubes, (**B**) ASPE-PM/g-C_3_N_4,_ and (**C**) magnified capture of g-C_3_N_4_ in PM nanonetwork. (**D**) General process and mechanism interaction of ASPE-PM/g-C_3_N_4_ with heavy metal ions in environmental water samples. Individual DPV response of the ASPE−PM/g-C_3_N_4_ composite-modified electrode of (**E**) Pb^2+^ and (**F**) Cd^2+^ over a concentration range of 0.1–1.0  µM. (**G**) Simultaneous DPV response of the ASPE-PM/g-C_3_N_4_ nanonetwork modified electrode of Pb^2+^and Cd^2+^ over a concentration range of 0.1–1.0  µM [110].

Conducting polymers (CPs), such as polyaniline (PANI), polypyrene (PPy), and poly(3,4-ethylenedioxythiophene) (PEDOT), have good electrochemical stability and high electrical conductivity, and are widely used in energy storage and electrochromic devices, ion sensing, and biosensors [159,160,161]. The excellent properties of polymer nanocomposites help promote the ability of electron transfer in electrochemical reactions, and they are an ideal material for improving the sensitivity of electrochemical detection [162,163]. In general, conducting polymers are not used alone but are compounded with other materials such as metals, metal oxides, and carbon materials. Wu et al. [106] compounded PEDOT and g-C_3_N_4_ to prepare a feasible method for the determination of heavy metal ions. The enhanced electrochemical performance of composite materials mainly depends on the strong synergy of the components. First of all, the use of the BCP method is conducive to the uniform combination of PEDOT and g-C_3_N_4_ flakes and strengthens the strong π–π interaction between them. Secondly, the fast charge transfer ability and large surface area of g-C_3_N_4_ promote the coordination of g-C_3_N_4_ with metal ions. In the work of Eswaran et al. [110], a new, low-cost, high-efficiency nano-engineered poly(melamine)/graphitic-carbon nitride nanonetwork (PM/g-C_3_N_4_)-modified screen-printed carbon electrode (SPE) for electrochemical monitoring of toxic HMIs in environmental water is proposed. Using the prepared PM/g-C_3_N_4_/ASPE as an environmental sensor, differential pulse voltammetry was used for selective and synchronous electrochemical detection of Pb^2+^ and Cd^2+^ ions. The sensor has good sensitivity and selectivity to Pb^2+^ and Cd^2+^, with sensitivities of 0.008 μM and 0.02 μM, respectively (Figure 12E–G). In addition, the composite of a D-A-D-conjugated polymer and g-C_3_N_4_ can be used as a good electrode modification material for constructing electrochemical sensors for the high-efficiency detection of heavy metal ions. In the work reported by Ding et al. [111], poly(2,5-bis(3,4-ethylenedioxythienyl)pyridine)/graphitic carbon nitride composites (poly(BPE)/g-C_3_N_4_) were prepared by an in situ chemical polymerization method and used for simultaneous detection of Cd^2+^ and Pb^2+^. The combination of poly(BPE) and g-C_3_N_4_ improves the conduction path on the electrode surface, and the conjugation effect between them enhances the adsorption of metal ions. In addition, the prepared modified electrodes can be used to detect Cd^2+^ and Pb^2+^ in tap water samples with recoveries of 98.64~106.74% and 99.81~113.15%, respectively. According to the report of Teng et al. [164], EDTA has been covalently immobilized onto CN-NS using *N*-(trimethoxysilylpropyl) ethylene-diamine triacetic acid sodium salt as a coupling reagent, which has been designed to improve the accumulation performance for Pb (II) onto the electrode surface. Studies have shown that the electrochemical response signal of Pb (II) on EDTA-CN-NS is significantly stronger than that of unfunctionalized CN-NS, and its LOD is 5.7 × 10^−13^ mol/L. The simultaneous electrochemical detection of Pb (II), Cu (II), and Hg (II) using the EDTA-CN-NS/Nafion/GCE was illustrated as well. In short, conductive polymer-modified graphitic carbon nitride nanocomposites have emerged as promising platforms for the electrochemical detection of heavy metal ions, leveraging the synergistic advantages of both components. Conductive polymers enhance the electrical conductivity, catalytic activity, and stability of g-C_3_N_4_, while the latter provides abundant active sites and tunable surface chemistry.

The doping of g-C_3_N_4_ is a widely studied strategy to enhance its physicochemical properties for applications in photocatalysis, energy storage, and environmental remediation. The effects of doping of different materials on the properties of g-C_3_N_4_ mainly include the following aspects:

(1) Enhanced photocatalytic performance

By introducing external dopant atoms into g-C_3_N_4_, the electronic structure and energy levels can be tuned to enhance photoresponsivity and improve charge separation. Non-metallic doping creates new energy levels within the band gap, which broadens the spectral response and reduces the electron–hole pair complexation rate. Therefore, through strategies such as non-metal doping, co-doping, and vacancy engineering, the photocatalytic performance of g-C_3_N_4_ can be enhanced by improving light absorption, facilitating charge separation and transport, and prolonging charge carrier lifetimes [165];

(2) Improved conductivity and charge transfer capability

The introduction of dopant atoms, on one hand, modifies the electronic structure of g-C_3_N_4_, significantly reducing the carrier migration barrier and thereby enhancing its intrinsic conductivity [166]. On the other hand, it creates additional active sites, introduces defects, and further improves conductivity;

(3) Improved thermal and chemical stability

Dopant atoms can be evenly dispersed in the C_3_N_4_ lattice as point defects, which can play a role similar to that of “pinning” and inhibit the expansion of lattice defects, thus improving the chemical stability of the material [167].

In summary, doping tailors g-C_3_N_4_’s properties by modifying its electronic structure, surface chemistry, and morphology, making it a versatile material for sustainable technologies. Future work should focus on optimizing dopant selection and understanding structure–property relationships at the atomic level.

## 5. Conclusions and Prospects

In environmental monitoring, electrochemical sensing methods are one of the most powerful tools. Generally, improving the stability and reliability of the electrochemical sensing interface requires a detailed investigation of the design and development of sensing materials. To achieve this goal, sensing materials need to have excellent adsorption and catalytic properties. In this feature article, we have presented an overview of g-C_3_N_4_ and g-C_3_N_4_-based nanocomposites: (1) characteristics specific for g-C_3_N_4_, (2) electrochemical detection of heavy metal ions, and (3) the application of g-C_3_N_4_ and g-C_3_N_4_-based nanocomposites in heavy metal detection. G-C_3_N_4_ has proven to be one of the most promising candidate materials for electrochemical sensing for designing and fabricating advanced composite catalysts for various applications.

Multifunctional materials will become a feasible direction for environmental safety monitoring in the future. In recent years, various nanomaterials such as precious metals, alloys, carbon materials, metal oxides, and their composites have been widely used in the electrochemical detection of heavy metal ions. Furthermore, in situ field analysis requires the development of portable integrated electrochemical devices and further advancement toward constructing highly integrated miniaturized electrochemical platforms. Currently, progress has been made in portable electrochemical sensors, where g-C_3_N_4_ nanocomposites combined with screen-printed electrodes have been preliminarily developed for environmental pollutant monitoring. Meanwhile, utilizing USB-powered electrochemical workstations and capsule-shaped electrochemical workstations configured with carbon electrodes, gold electrodes, and graphene electrodes modified with g-C_3_N_4_ nanocomposites enables the detection of specific biomarkers. The detection signals can be wirelessly transmitted via Bluetooth to mobile phones or other display terminals, demonstrating promising potential for real-time monitoring of toxic contaminants in aquatic environments.

Though a large number of studies have been devoted to this field and some significant results have been achieved, some important issues are still worth studying. Firstly, there are still some problems to be solved in the large-scale preparation of g-C_3_N_4_ with controllable morphology. Current synthesis methods for g-C_3_N_4_ nanostructures (e.g., quantum dots and porous frameworks) often lack reproducibility and scalability. Standardized protocols and cost-effective fabrication techniques (e.g., green chemistry approaches and roll-to-roll manufacturing) are urgently needed for industrial adoption. Secondly, complex environmental matrices (e.g., organic matter and competing ions) often compromise sensor selectivity and accuracy. Developing surface modification strategies or selective recognition elements (e.g., aptamers and ion-imprinted polymers) is essential to enhance anti-interference capabilities. Lastly, balancing ultra-trace detection limits (sub-ppb) with high specificity in multi-ion systems remains challenging, necessitating advanced signal amplification mechanisms or machine learning-assisted data processing. Therefore, future efforts should prioritize surface engineering, interdisciplinary integration (e.g., AI-guided material design), and pilot-scale validation to bridge the gap between laboratory research and practical environmental monitoring applications.

Furthermore, g-C_3_N_4_-based sensors offer high sensitivity and selectivity for HMIs, but challenges in scalability, stability, and device integration hinder commercialization. For example, high-quality g-C_3_N_4_ requires complex synthesis steps, such as thermal polymerization and exfoliation, which are energy-intensive. Therefore, the production of defect-free nanosheets on an industrial scale remains challenging; prolonged exposure to harsh electrochemical conditions (e.g., acidic media) can degrade g-C_3_N_4_ composites, reducing sensor lifespan; most g-C_3_N_4_ sensors rely on glassy carbon electrodes (GCEs). Adapting them to wearable or field-deployable formats requires miniaturization and compatibility with flexible substrates, which are underdeveloped. Based on this, innovations can be made in material design (such as defect engineering and hybrid composite materials) and synthesis methods (such as microwave-assisted routes), striving to accelerate the transition from lab-scale prototypes to market-oriented devices.

## Figures and Tables

**Table 1:** Comparison of the determination performance of electrochemical sensors based on graphite phase carbon nitride in the determination of heavy metal ions.

Electrodes	HMIs	Methods	Range of Linearity (μM)	Sensitivity (μA·μM^−1^)	LOD (μM)	Ref.
g-C_3_N_4_/GC	Pb(II)	SWASV	0.5~3.5	10.887	0.228	[87]
	Cu(II)		0.5~4.5	4.794	0.103	
	Hg(II)		0.5~4.5	18.180	0.217	
g-C_3_N_4_/Nafion/GC	Cd(II)	DPASV	0.001~100	/	0.5 × 10^−3^	[88]
g-C_3_N_4_ nanosheet/GC	Cd(II)	SWASV	0.05~0.7	22.668	3.94 × 10^−3^	[24]
Ultrathin g-C_3_N_4_/GC	Cd(II)	SWASV	0.001~0.1	43.9	0.35 × 10^−3^	[72]
g-C_3_N_4_/Pt/PAn NCs/GC	Hg(II)	DPASV	0.5~1	1.0787	9 × 10^−3^	[89]
g-C_3_N_4_ nanosheet/GC	Pb(II)	DPASV	0.0075~1.0	33.08	1 × 10^−3^	[90]
S-g-C_3_N_4_/GC	Pb(II)	DPV	0.075~2.5	0.220	3 × 10^−3^	[91]
Au/g-C_3_N_4_/rGO/GC	Pb(II)	DPASV	0~0.1	0.618	0.1 × 10^−3^	[92]
AuNPs/mpg-C_3_N_4_/GC	Cr(VI)	LSV	0~19.3	0.110	0.283	[7]
AuNPs/mpg-C_3_N_4_/GC	MeHg(I)	DPASV	0~0.116	61.4	0.47 × 10^−3^	[93]
S-g-C_3_N_4_/Au electrode	MeHg(I)	DPASV	0~0.116	110.0	0.81 × 10^−3^	[94]
S-g-C_3_N_4_ tube bundles/graphene nanosheets	Pb(II)	SWASV	0.025~8.5	21.684	0.78 × 10^−3^	[95]
	Hg(II)		0.05~7.5	15.71	1.15 × 10^−3^	
	Cd(II)		0.05~5	10.132	2.30 × 10^−3^	
g-C_3_N_4_@FeP-C/GC	Cu(II)	DPASV	0.05~20	0.125	0.0167	[96]
g-C_3_N_4_/CNT/NH_2_-MIL-88(Fe)/GC	Cd(II)	SWSV	0.12~6.0	3.66	39.6 × 10^−3^	[97]
	Pb(II)		0.02~6.0	19.15	7.6 × 10^−3^	
	Cu(II)		0.04~6.0	12.15	11.9 × 10^−3^	
	Hg(II)		0.03~6.0	15.10	9.6 × 10^−3^	
rGO/g-C_3_N_4_/GC	Pb(II)	SWASV	0.00001~1	195.22	1.07 × 10^−6^	[98]
Au/N-deficient-g-C_3_N_4_/GC	Pb(II)	SWASV	0.2~0.8	184.4	29 × 10^−3^	[99]
g-C_3_N_4_/rGO/GC	Pb(II)	SWASV	0.05~1.45	13.6	0.72 × 10^−3^	[100]
CuS/g-C_3_N_4_/GC	Pb(II)	SWASV	0.05~5.000	10.811	4 × 10^−3^	[101]
Bi/g-C_3_N_4_/SPE	Pb(II)	SWASV	0.267~1.067	4.98	0.156	[23]
	Cd(II)		0.145~0.531	1.787	0.039	
Ti_3_C_2_(HF)/Fe_3_O_4_/g-C_3_N_4_/GC	Pb(II)	DPASV	0.005~0.5	57.23	0.12	[102]
	Cd(II)			44.14		
	Hg(II)			48.24		
Pd/g-C_3_N_4_/GC	Hg(II)	DPV	0.05 × 10^−3^~0.025	42.281	0.45 × 10^−3^	[103]
Fe_2_O_3_/g-C_3_N_4_/GC	Pb(II)	PEC	3.0 × 10^−4^~4.8	40.6	3.8 × 10^−5^	[104]
g-C_3_N_4_/CNT/NH_2_-MIL-88(Fe)/GC	Pb(II)	SWSV	0.02~6.00	19.15	7.6 × 10^−3^	[97]
	Cu(II)		0.04~6.00	12.15	11.9 × 10^−3^	
	Hg(II)		0.03~6.00	15.10	9.6 × 10^−3^	
	Cd(II)		0.12~6.00	3.66	39.6 × 10^−3^	
pg-C_3_N_4_/CoMn_2_O_4_/GC	Pb(II)	SWASV	0.2~4.4	1.660	1.4 × 10^−3^	[105]
	Cd(II)		0.5~7.0	5.178	2.1 × 10^−3^	
Pt/g-C_3_N_4_/Polythiophene/GC	Hg(II)	DPV	0.01~5	76.15	9 × 10^−6^	[89]
PEDOT/g-C_3_N_4_/GC	Pb(II)	DPV	0.04~11.6	8.8135	4.21 × 10^−3^	[106]
	Cd(II)	DPV	0.06~12	3.0645	1.4 × 10^−3^	
Porous-g-C_3_N_4_/O-MWCNTs/SPE	Pb(II)	DPV	0.17 × 10^−3^~0.53	/	0.038 × 10^−3^	[107]
	Cd(II)		0.038~2.233	/	0.266 × 10^−3^	
	Zn(II)		0.064~3.089	/	0.917 × 10^−3^	
	Hg(II)		0.024~0.459	/	0.199 × 10^−3^	
g-C_3_N_4_/CB composite electrode	Cd(II)	DPASV	0~0.7	11.0	2.1 × 10^−3^	[81]
	Pb(II)		0~0.3	9.0	0.26 × 10^−3^	
	Hg(II)		0~0.5	4.0	0.22 × 10^−3^	
g-C_3_N_4_/Ti_3_C_2_T_x_/GC	Cd(II)	SWASV	0.05~1.5	40.97	1 × 10^−3^	[108]
	Pb(II)		0.05~1.5	49.91	0.6 × 10^−3^	
P-CN_T60/MWCNT/CFE	Cu(II)	DPSV	6.6 × 10^−6^~8.5	0.094	1.0 × 10^−7^	[109]
	Pb(II)		0.81 × 10^−3^~8.5	0.030	1.8 × 10^−5^	
	Hg(II)		0.22 × 10^−6^~8.5	0.021	8.0 × 10^−6^	
poly(melamine)/g-C_3_N_4_/SPE	Pb(II)	DPV	0.1~1	/	8 × 10^−3^	[110]
	Cd(II)		0.1~1	/	20 × 10^−3^	
poly(BPE)/g-C_3_N_4_/GC	Cd(II)	DPV	0.12~7.2	7.636	9.7 × 10^−3^	[111]
	Pb(II)		0.08~7.2	11.140	3.27 × 10^−3^	

## Data Availability

No new data were created or analyzed in this study.
